# Probing the Surface Chemistry of Nanoporous Gold via Electrochemical Characterization and Atom Probe Tomography

**DOI:** 10.3390/nano11041002

**Published:** 2021-04-14

**Authors:** AmirHossein Foroozan-Ebrahimy, Brian Langelier, Roger Charles Newman

**Affiliations:** 1 Corrosion and Advanced Materials Laboratory, Department of Chemical Engineering and Applied Chemistry, University of Toronto, 200 College Street, Toronto, ON M5S 3E5, Canada; roger.newman@utoronto.ca; 2 Canadian Centre for Electron Microscopy, Department of Materials Science and Engineering, McMaster University, 1280 Main Street West, Hamilton, ON L8S 4L8, Canada; langelb@mcmaster.ca

**Keywords:** nanoporous gold, electrochemical characterization, cyclic voltammetry, sulfur adsorption, atom probe tomography, dealloying

## Abstract

Surface chemistry information is crucial in understanding catalytic and sensing mechanisms. However, resolving the outermost monolayer composition of metallic nanoporous materials is challenging due to the high tortuosity of their morphology. In this study, we first elaborate on the capabilities and limitations of atom probe tomography (APT) in resolving interfaces. Subsequently, an electrochemical approach is designed to characterize the surface composition of nanoporous gold (NPG), developed from dealloying an inexpensive precursor (95 at. % Ag, 5 at. % Au), by the means of aqueous electrochemical measurements of the selective electrosorption of sulfide ions, which react strongly with Ag, but to a significantly lesser extent with Au. Accordingly, cyclic voltammetry was performed at various scan rates on NPG in alkaline aqueous solutions (0.2 M NaOH; pH 13) in the presence and absence of 1 mM Na_2_S. Calibrations via similar voltammetric measurements on pure polycrystalline Ag and Au surfaces allowed for a quantitative estimation for the Ag surface coverage of NPG. The sensitivity threshold for the detection of the adsorbate–Ag interaction was assessed to be approximately 2% Ag surface coverage. As curves measured on NPG only showed featureless capacitive currents, no faradaic charge density associated with sulfide electrosorption could be detected. This study opens a new avenue to gain further insight into the monolayer surface coverage of metallic nanoporous materials and assists in enhancement of the interpretation of APT reconstructions.

## 1. Introduction

Nanoporous gold (NPG) derived from selective electrolytic dissolution of Ag from Ag–Au(-Pt) alloys [1,2] has shown promising results in terms of catalysis, sensing, bioanalytical and biomedical applications [3,4,5,6,7]. Gaining insight into the surface composition of metallic nanoporous materials could not only enhance the understanding of the underlying mechanisms of nanoporosity evolution and catalysis, but would be crucial in biosensing applications, in particular, in vivo sensors. However, obtaining insight into the entire surface composition of such tortuous nanostructures has proved to be challenging despite the use of various advanced atomic-scale characterization techniques such as atom probe tomography (APT) [8,9]. While APT reconstructions describe the materials with an atomic resolution, the composition of monolayers (subnanometer bins) cannot be resolved with certainty due to APT aberrations [10,11,12]. Additionally, APT is a destructive and highly localized method of investigation, hence there is a need for a non-local and non-destructive surface analysis method to investigate the surface composition of such nanoporous materials.

This study is an attempt at shedding further light on the surface composition of NPG by augmenting APT data with aqueous electrochemical techniques. The question of how much of the surface of the NPG is still covered by Ag is raised and electrochemical characterization is employed for the investigation.

It is hypothesized that measuring electrochemical responses from selective interactions of certain species with the surface of NPG advances the analysis of the composition of its outermost monolayer. The most straightforward electrochemical approach to this problem would be a comparison of electrosorption of aqueous ions that would react readily with one element on the surface but would react to a significantly lesser extent with the other element on the surface; or ideally would not react at all. Assuming that this difference in reactivity could be captured electrochemically, a quantitative assessment regarding the surface composition of electrodes could potentially be obtained.

Electrochemical surface characterization has already been used to estimate the platinum surface coverage of NPG structures developed from electrochemical dealloying of Ag–Au–Pt alloy precursors containing 20 to 23 at. % Au and a small atomic percentage of Pt (1 to 3 at. %) [13]. The electro-adsorption of under-potentially deposited hydrogen (H_UPD_) is characteristic of Pt group metals only, while over-potential deposition of hydrogen occurs on all electrode materials during the hydrogen evolution reaction [14]. Measurements of the charge density for H_UPD_ on NPG structures (formed by dealloying Ag–Au–Pt alloys) and their comparison to the values reported in the literature for pure polycrystalline Pt surfaces [15] have allowed Pt surface coverage estimations. Such estimations have showed surprisingly good agreement with surface analysis results from X-ray photoelectron spectroscopy (XPS) [13,16]. However, whether isolated Pt atoms on the surface would interact with hydrogen, and in general the ensemble effects of Pt in H_UPD_ on alloys are not fully elucidated yet [17,18,19,20].

In this study we leverage the selective electrosorption of hydrosulfide ions (HS^−^) on Ag as measured via cyclic voltammetry to characterize the surface composition of NPG developed from electrolytic dealloying of lean noble alloys containing only 5 at. % Au (Ag_95_Au_5_).

## 2. Materials and Methods

### 2.1. Sample Preparation

A 99.999% pure gold wire (purchased from Alfa Aesar, Tewksbury, MA, United States) with a diameter of 1 mm was spot welded to a copper wire. The junction was then masked using Miccroshield lacquer (purchased from Structure Probe, Inc., West Chester, PA, United States) resulting in an exposed surface area of 0.17 cm^2^. The wire was cleaned in 20% nitric acid followed by rinsing with copious amount of deionized (DI) water prior to each measurement.

A 99.999% pure silver rod (purchased from Alfa Aesar, Tewksbury, MA, United States) with a diameter of 7 mm (a geometric surface area of 0.38 cm^2^) was mounted in epoxy and polished with abrasive paper down to 800 grit finish. Then, the surface was cleaned ultrasonically in ethanol and was finally rinsed with DI water and dried with pressurized air before each experiment.

Silver–gold alloys were fabricated using a vertical induction cold crucible semi-levitation furnace custom designed by and purchased from Arcast Inc (Oxford, Maine, United States). A total of 5 g of Ag–Au binary alloy containing 5 at. % gold, balanced with silver (Ag_95_Au_5_), which corresponds to 91.24 wt. % Ag and 8.76 wt. % Au was fabricated using 99.999% pure Au and Ag pellets purchased from Kurt J. Lesker Company (East Sussex, United Kingdom). The melting process was done twice to ensure perfect mixing. The resulting lumps of alloys were then cut into 1 mm sheets using a Buehler (Lake Bluff, IL, United States) IsoMet 1000 Precision Cutter equipped with a diamond blade. The sheets were cold rolled into 200 µm foils. Small samples ranging from 0.1 to 0.5 cm^2^ were then cut out of the foils. Annealing was performed in H_2_-Ar atmosphere (2.5% H_2_, balanced with Ar) at 900 °C for 5 h. All the alloy specimens were used in the as-annealed condition without any further surface preparation. In preparation for the electrochemical measurements, the samples were spot welded to copper wires. The junction was then masked using lacquer.

### 2.2. Electrochemistry

All electrochemical measurements were performed using a Gamry (Warminster, PA, United States) Reference 600 digital potentiostat controlled by Gamry Instruments Framework software. The charges associated with electrosorption measured by cyclic voltammetry were calculated using Gamry Echem Analyst version 5.60.

All solutions were prepared using reagent grade de-ionized water (Type 1, 18.2 MΩ cm resistivity) and were deaerated for 30 min by high-purity nitrogen (minimum purity: 99.998%) purging via a fine porous glass frit prior to the onset of the experiments. During the electrochemical measurements, the deaeration was continued at a lower flow rate above the level of the solution to eliminate the possibility of oxygen intake. A Pt foil with a surface area of 2 cm^2^ was used as a counter electrode.

#### 2.2.1. Cyclic Voltammetry

Cyclic voltammetry was performed at scan rates of 20, 50 and 100 mV s^−1^ to investigate sulfur adsorption/desorption on the surfaces. Measurements on Ag and Au electrodes were used to calibrate the peaks as a baseline for the characterization of the surface of as-annealed Ag_95_Au_5_ alloy and NPG electrodes.

A basic solution (pH 13) of 0.2 M sodium hydroxide (NaOH) with and without 1 mM sodium sulfide (Na_2_S) was used for cyclic voltammetry experiments. Both chemical compounds used in these experiments were Analar grade and purchased from Sigma Aldrich (St. Louis, MO, United States). The deaeration step preceded Na_2_S addition, which was followed by vigorous stirring by a magnetic stir bar.

The reference electrode (RE) used in 0.2 M NaOH solutions was a mercury/mercury oxide electrode with a 20% potassium hydroxide (4.24 M KOH) electrolyte (Hg/HgO; +0.09 V vs. standard hydrogen electrode (V_SHE_)). For the electrochemical measurements in the presence of 1 mM Na_2_S, a silver/silver sulfide (Ag/Ag_2_S) RE was fabricated by anodizing a high-purity (99.99%) Ag wire (with a diameter of 1 mm and a length of 7 cm) at +0.5 V vs. a saturated calomel electrode (SCE; +0.24 V_SHE_) for 30 s in a 1 M Na_2_S solution [21]. Measurements against Hg/HgO RE for an extended period of time revealed a stable potential at −0.64 V for the Ag/Ag_2_S RE.

The upper limit of the scanned potential window was chosen at −0.07 V_Ag/Ag2S_ to avoid bulk Ag sulfidation, which in the presence of Na_2_S would result in Ag_2_S film formation and consequent surface roughening after several redox cycles [22]. The consideration with the lower potential limit (−0.65 V_Ag/Ag2S_) was to avoid large current densities for hydrogen evolution, for which the equilibrium potential is approximately −0.8 V_SHE_ according to the Nernst equation at the conditions of the experiments.

#### 2.2.2. Electrochemical Dealloying

A 250 mL electrochemical cell containing 125 mL 0.5 M HClO_4_ solution, prepared from Analar grade HClO_4_ (62%, Alfa Aesar, Tewksbury, MA, United States) was employed for dealloying experiments. A mercury/mercury sulfate electrode (MSE; +0.64 V_SHE_) was used as the reference electrode. Dealloying was done via potentiostatic chronocoulometry at 0.3 V_MSE_ and to a charge density of 2.5 C cm^−2^. In all cases, nanoporosity was induced on all sides of the samples.

Upon dealloying, samples were removed from the cell and immediately washed with DI water, followed by immersion in a large volume of DI water for 10 min. Then, cyclic voltammetry measurements were performed immediately after rinsing with DI water and open-circuit potential (OCP) stabilization (~20 min) in the fresh electrolytes.

#### 2.2.3. Electrochemical Impedance Spectroscopy (EIS)

To quantify the increase in the surface area after dealloying, and to gauge the appropriateness of the scan rates used in voltammetry of NPG electrodes, double-layer capacitance measurements were employed using EIS at OCP, AC voltage 10 mV rms, frequency range 100 kHz–0.1 Hz with 10 points/decade.

## 3. Results

### 3.1. APT Limitation in Resolving Interfaces

**Figure 1:**
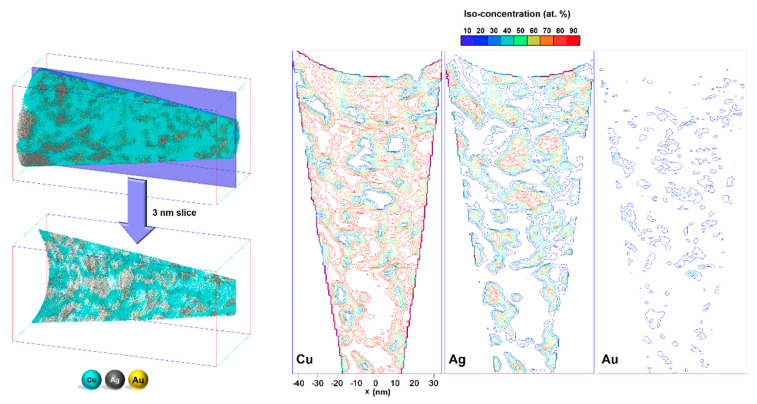
A 3 nm slice of the Cu-filled NPG developed from Ag_94_Au_5_Pt_1_ alloy (for detailed dealloying and APT procedures please refer to [9]) with different iso-concentration contour lines, color-coded for different at. % Cu, Ag, and Au values of 10 to 90%.

To enhance the mechanical properties of the dealloyed materials in preparation for APT, Cu electrodeposition is used to fill the nanoporosity [8]. The differences in chemistry and structure of Cu-filled NPG samples impart surface imperfections which affect the electronic field projecting from the tip of the APT sample, which in turn leads to trajectory aberrations in the departing ions, and can cause both local magnification or de-magnification of phases, as well as overlap of atoms from different phases in the APT reconstructions. The effect of these local evaporation artifacts on the position of measured atoms is illustrated in Figure 1. As revealed by the plotted iso-concentration contours, Cu—originally absent from the NPG phase—is detected throughout almost all regions of the dataset due to the prevalence of trajectory aberrations. Although established APT data analysis algorithms, such as proximity histograms and surface excess calculations, can yield insightful information about the overall composition and shape of the ligaments [8,9,23,24,25], the local evaporation artifacts prevent an atomic-scale look at the monolayer covering the ligament surfaces.

It is noteworthy to mention that the viability of forming nanoporosity from such lean noble alloys has been debated [26,27]. For a given average ligament diameter, the theoretical minimum threshold of the Au content in binary Ag–Au precursor alloys required to render the formation of a fully Au covered nanoporous morphology after dealloying can be estimated. Modeling the ligaments as Au covered cylinders with Ag cores suggests a minimum Au content of about 5 at. % for an average ligament size of ca. 15 nm [9].

### 3.2. Electrochemical Characterization

**Figure 2:**
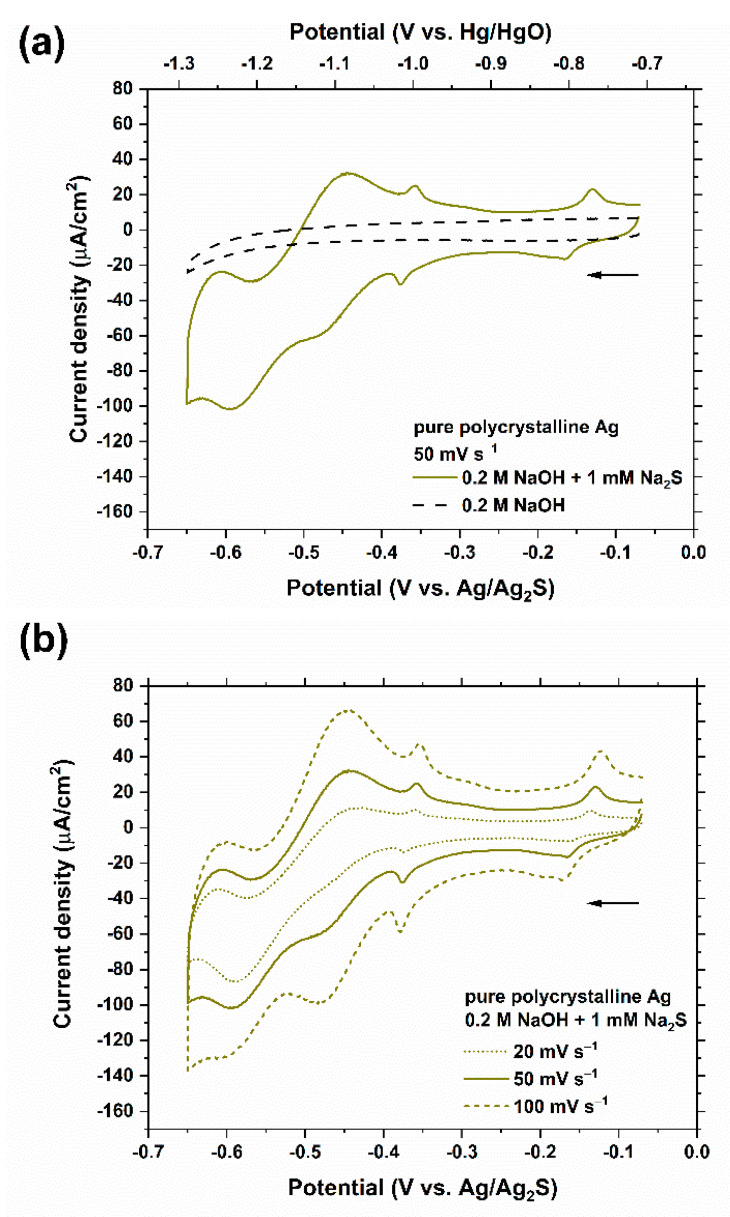
Cyclic voltammetry of a polycrystalline Ag electrode (0.38 cm^2^) in 0.2 M NaOH solution with and without 1 mM Na_2_S (**a**) at a scan rate of 50 mV s^−1^; (**b**) and in the presence of 1 mM Na_2_S at different scan rates (20, 50, 100 mV s^−1^). The arrow indicates the starting point and the scan direction.

Cyclic voltammograms for a pure polycrystalline Ag electrode in 0.2 M NaOH solution in the presence and absence of 1 mM Na_2_S are shown in Figure 2. The voltammetric curves obtained in solutions containing only 0.2 M NaOH (Figure 2a) show a featureless capacitive current except for the region where the hydrogen evolution reaction (HER) is kinetically favorable. In the solutions containing 1 mM Na_2_S, however, three partially overlapping peaks and an isolated peak are present at −0.60, −0.47, −0.37, and −0.15 V_Ag/Ag2S_ half-wave potentials (E_1/2_ = (E_pc_ + E_pa_)/2 where E_pc_ and E_pa_ represent the cathodic and anodic peak potentials, respectively), respectively, and are in excellent agreement with the values reported in the literature for sulfur adsorption/desorption potentials in similar systems [28,29,30,31,32].

For solutions containing Na_2_S in this investigation, the exclusive sulfur species present in the alkaline electrolyte used (0.2 M NaOH, pH 13) is hydrosulfide (HS^−^) as determined from the literature pKa values for H_2_S (7.02) and HS^−^ (17.1) [33] and potential-pH equilibrium diagrams for the S-H_2_O system at room temperature [34]. Moreover, similar voltammetric measurements using ^35^S-labeled Na_2_S and radiotracer techniques [35] have revealed that the adsorption of HS^−^ (Equation (1)) occurs within the potential range encompassing the first three peaks (at −0.60, −0.47, and −0.37 V_Ag/Ag2S_) shown in Figure 2.

Ag + HS^−^ = AgSH_ads_ + e^−^ (1)

The appearance of more than one peak suggests that the HS^−^ adsorption is more complex than is indicated in Equation (1), perhaps involving cation coadsorption and/or potential dependent structural rearrangement of the adlayer [29]. Generally, multiple cycles of voltammetric measurements on a number of electrodes indicated a high level of reproducibility for the observed peaks.

The well-resolved peak at −0.15 V_Ag/Ag2S_ is attributed to the oxidation of AgSH_ads_ into Ag_2_S_ads_ (Equation (2)) [29,30,35]. This step does not correspond to a significant increase in the sulfur surface coverage, but instead, results from an oxidative phase transition involving a pre-existing and essentially complete AgSH_ads_ layer [29].

Ag + AgSH_ads_ + OH^−^ = Ag_2_S_ads_ + H_2_O + e^−^ (2)

**Figure 3:**
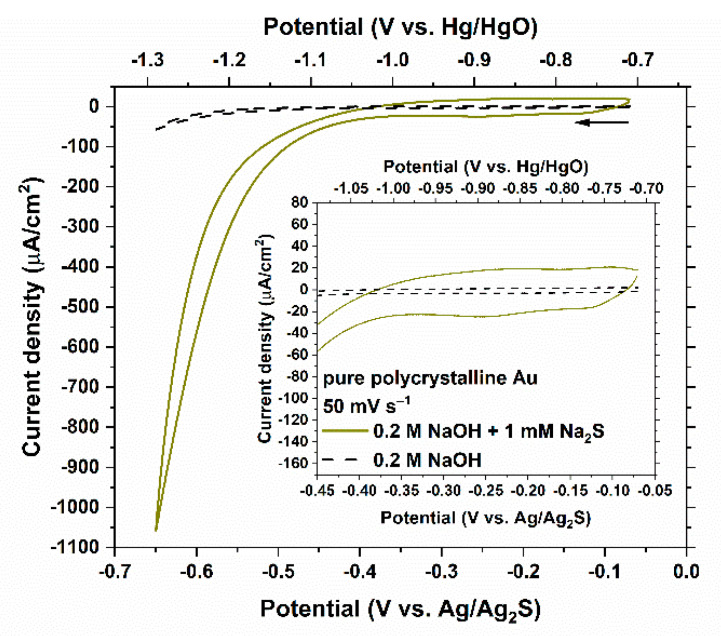
Cyclic voltammetry of a polycrystalline Au wire (0.17 cm^2^) in 0.2 M NaOH solution with and without 1 mM Na_2_S at a scan rate of 50 mV s^−1^. The insert shows a more refined scale. The arrow indicates the starting point and the scan direction.

Similar measurements on a pure polycrystalline Au electrode are shown in Figure 3 and with a more refined scale depicted in the insert. The behavior of Au in 0.2 M NaOH solution is purely capacitive within the potential range where the HER does not interfere. No distinct peak can be resolved in the presence of 1 mM Na_2_S, which attests to the weak electrochemical interactions between Au and HS^−^ as compared to those with the surface of Ag. Although there are reports of kinetically sluggish sulfur adsorption peaks on Au in different aqueous systems than the ones used in this study [36], there are various contrasting results among different investigators [37,38,39,40,41]. The absence of any conspicuous peak in the cyclic voltammetry of Au in the Na_2_S containing electrolyte used in this study indicates that HS^−^ is suitable for the purpose of surface characterization of NPG.

The current densities at potentials more negative than −0.4 V_Ag/Ag2S_ are considerably affected by the HER (Figure 2 and Figure 3). Therefore, for the surface characterization discussion, only the portions of the curves that are located more positive than −0.4 V_Ag/Ag2S_ were considered. The peak observed at −0.37 V_Ag/Ag2S_ in the case of the Ag electrode was selected as the electrochemical signal that distinguishes Ag from Au since the HER contributions to the current densities around this potential are negligible on both electrodes and the behavior of Au appears to be purely capacitive.

After integrating the areas below the curves corresponding to the HS^−^ adsorption/desorption on pure Ag in 0.2 M NaOH containing 1 mM Na_2_S, within the potential range where the peak at −0.37 V_Ag/Ag2S_ is present—between −0.255 and −0.385 V_Ag/Ag2S_—and averaging the values for both anodic and cathodic peaks observed at scan rates of 20, 50, and 100 mV s^−1^, and subtracting the charge density corresponding to the curves measured in the absence of 1 mM Na_2_S, a total faradaic charge density of 30.39 ± 2.01 µC cm^−2^ was calculated. The plus-minus value represents the standard deviation among two sets of measurements.

**Figure 4:**
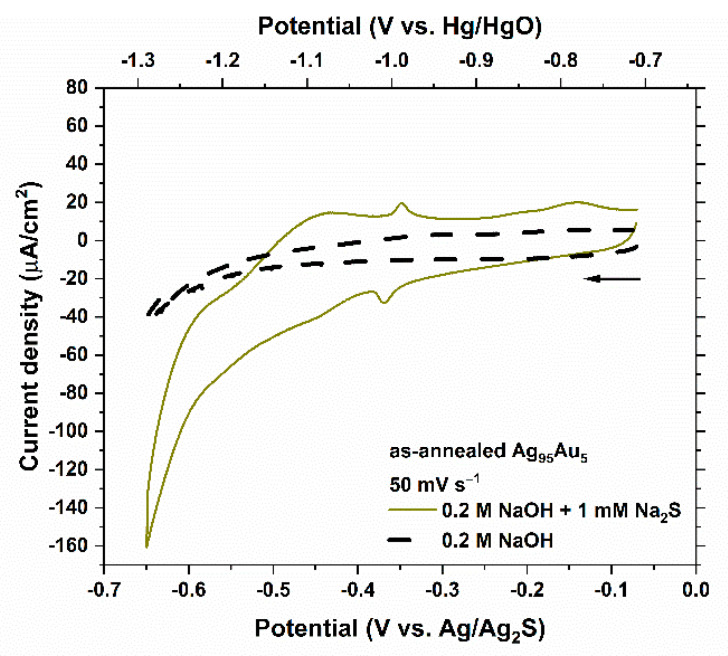
Cyclic voltammetry of an as-annealed (non-dealloyed) Ag_95_Au_5_ alloy (0.25 cm^2^) in 0.2 M NaOH solution with and without 1 mM Na_2_S at a scan rate of 50 mV s^−1^. The arrow indicates the starting point and the scan direction.

Figure 4 shows the cyclic voltammetry measurements conducted on the surface of as-annealed (non-dealloyed) Ag_95_Au_5_ alloy. Since the metallic radii of Ag and Au are virtually the same, 95% of the surface area of Ag_95_Au_5_ alloy can be regarded as Ag, assuming negligible surface segregation during the annealing stage [42].

Due to the lower free surface energy and molar heat of sublimation of Ag compared to Au, Ag atoms in a solid solution of Ag–Au alloy are expected to preferentially diffuse and concentrate at the surface. Several studies have verified Ag surface segregation in Ag–Au systems under ultra-high vacuum (UHV) conditions [43,44,45,46] and under various oxidative atmospheres [47,48,49,50]. A recent APT investigation of surface segregation in Ag–Au alloys at elevated temperatures (323 K < T < 673 K) indicated that under an unreactive atmosphere, such as Ar (6 kPa), Ag segregation is negligible for short treatment duration (1 h) [42]. On the other hand, reductive treatments in H_2_ was found to lead to Au enrichment on the surface [42]. Overall, given the annealing atmosphere of 2.5% H_2_ balanced with Ar at 1 atm used for the Ag_95_Au_5_ samples in our study, negligible surface segregation is a safe assumption, as also demonstrated by electrochemical characterization in the following.

The four peaks associated with the HS^−^ adsorption/desorption on pure Ag (Figure 2b) appear dampened—each to a different extent—in Figure 4. An average value of 28.40 ± 1.08 µC cm^−2^ was yielded for the total faradaic charge density of the adsorption/desorption of HS^−^ on the surface of as-annealed Ag_95_Au_5_ within the potential range of −0.255 and −0.385 V_Ag/Ag2S_ using the same method described earlier in the case of pure Ag electrode. This value is in a good agreement with what would be expected based on the calibration with pure Ag electrode (0.95 × (30.39 ± 2.01) = 28.87 ± 1.91 µC cm^−2^).

**Figure 5:**
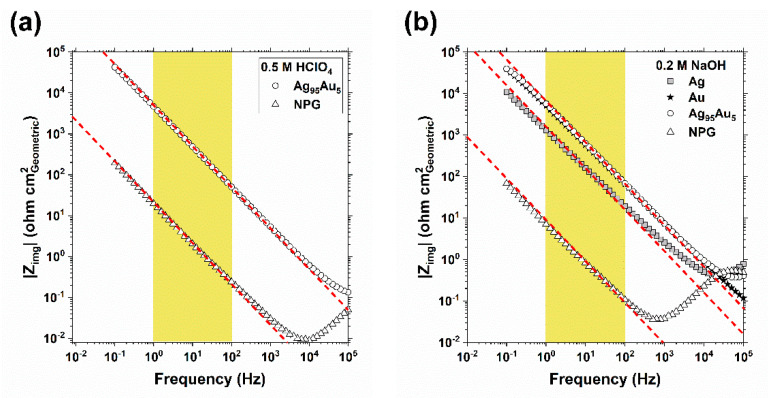
The dependence of the absolute value of the imaginary component of impedance (|Z_img_|) against frequency measured by EIS at OCP in (**a**) 0.5 M HClO_4_; and (**b**) in 0.2 M NaOH for NPG and on the flat surfaces of Ag, Au, and Ag_95_Au_5_ electrodes. The red dashed lines indicate a slope of −1.

To normalize the current response of the cyclic voltammetry measurements on the NPG samples by their true surface area, electrochemical impedance spectroscopy (EIS) measurements were performed before and after dealloying in deaerated 0.5 M HClO_4_ solution (Figure 5a) to estimate the double-layer capacitance (C_dl_) using the relationship presented in Equation (3).

C_dl_ = (2πf|Z_img_|)^−1^ (3)

Then, the ratio of the C_dl_ after dealloying to the C_dl_ before dealloying as measured at 10 Hz, which is ca. 240 for dealloying this particular Ag_95_Au_5_ sample to 2.5 C cm^−2^, was used as a measure of the factor by which the surface area has been multiplied as a result of nanoporosity evolution, assuming that the surface composition change does not change the elementary capacitance.

Estimating the surface area of NPG using C_dl_ measurement via EIS has shown an excellent correlation with the results from the Brunauer–Emmett–Teller (BET) technique [13]. Comparisons between the EIS estimations of the C_dl_ of NPG and estimations made by other electrochemical techniques have indicated that the surface area estimated by EIS is 1.5- to 1.75-fold larger than estimations made by Cu UPD or Au oxidation/reduction methods [51]. However, the effects of post-porosity coarsening during measurements involving polarization of the NPG electrodes have not been considered.

A thorough investigation into the post-porosity coarsening of NPG developed from lean noble alloys is the subject of a future study. Preliminary observations indicated that after 1 h of exposure to 0.5 M HClO_4_ and simultaneous polarization at 0 V_MSE_, which is well below the critical potential (+0.125 V_MSE_; Figure 2 in [9]), the surface area reduced by a factor of ca. 0.9, as measured via EIS by the method described earlier. Since Cu UPD and Au oxidation/reduction methods require polarization of the electrodes, we have adhered to EIS at OCP to estimate the true surface areas in this study.

The dependence of the absolute value of the imaginary impedance (|Z_img_|) on frequency (f) for the NPG, Ag_95_Au_5_, Ag, and Au samples in 0.2 M NaOH is displayed in Figure 5b. The plot of log(|Z_img_|) vs. log(f) has a constant slope of −1 for an ideal capacitive behavior. Over at least two frequency decades, from 1 to 100 Hz (highlighted with yellow), the slope of the plots is close to −1 (red dashed lines), suggesting a nearly ideal capacitive behavior. Figure 5b indicates that the dependence of the C_dl_ estimations on f for the NPG sample is similar to those for flat Ag_95_Au_5_, Ag, and Au surfaces over a wide range of frequencies.

**Figure 6:**
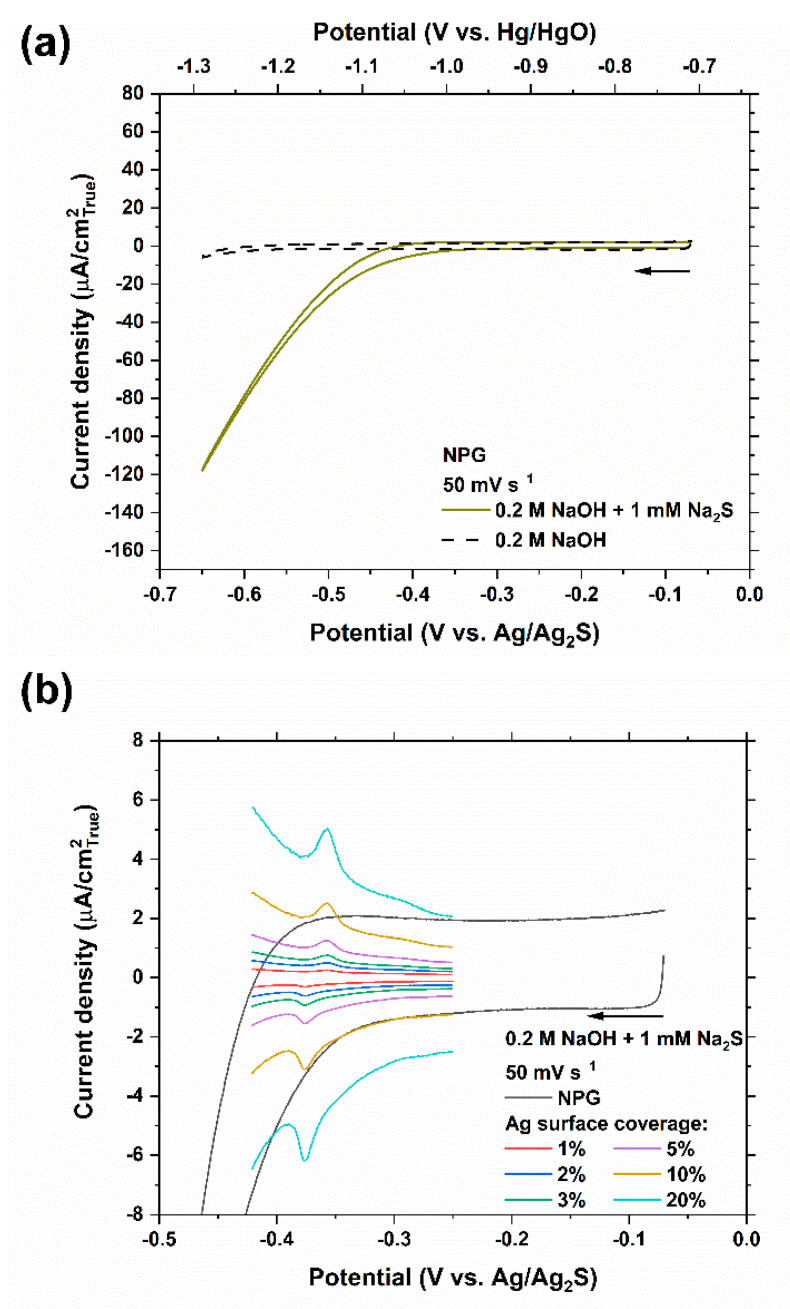
(**a**) Cyclic voltammetry of NPG (from dealloying of as-annealed Ag_95_Au_5_ in 0.5 M HClO_4_ at 0.3 V_MSE_ to 2.5 C cm^−2^) in 0.2 M NaOH solution with and without 1 mM Na_2_S at a scan rate of 50 mV s^−1^. True surface areas, as estimated by EIS at OCP in 0.5 M HClO_4_ (Figure 5a), have been used to calculate the current densities. (**b**) A more refined scale of (**a**) superimposing the HS^−^ electrosorption peak observed at −0.37 V_Ag/Ag2S_ on pure Ag electrode adjusted in current density to represent different Ag surface coverages. The arrow indicates the starting point and the scan direction.

Cyclic voltammetries on NPG electrodes show featureless curves (Figure 6). The measurement on NPG in the presence of 1 mM Na_2_S is also shown in Figure 6b with a finer scale and the superimposition of the HS^−^ electrosorption peak observed at −0.37 V_Ag/Ag2S_ on pure Ag electrode adjusted in current density to represent various Ag surface coverages. The peaks can be distinguished from the capacitive current density on Ag surface coverages as low as 2%. The ensemble effects of Ag in HS^−^ electrosorption on binary Ag–Au systems can be examined in further work by analyzing the results from model alloys and fabricated surfaces.

On a separate note, the overpotential required for HER is considerably lower in the 0.2 M NaOH solutions containing 1 mM Na_2_S on the surface of pure Au, as seen in Figure 3, and on the Au-enriched surface of NPG, as seen in Figure 6. It is more difficult to discern this trend from the measurements on pure Ag or as-annealed Ag_95_Au_5_ alloy due to the interference from the current densities associated with the oxidation of AgSH_ads_ into Ag_2_S_ads_. There are also reports of enhanced proton and water reduction rates on heterogeneous catalysts composed of noble and/or transition metal sulfides [52,53,54,55,56].

## 4. Conclusions

New insight into the composition of the outermost monolayer of a nanoporous metallic material was gained by designing an aqueous electrochemical method for surface characterization, based on selective and reversible electrosorption of anions on the electrode.

The question of to what extent the outermost monolayer of a nanoporous structure, formed from dealloying lean noble alloys, is composed of the more noble element was raised. Limitations of APT in resolving the surface composition of the NPG were elucidated. Electrochemical methods were adopted to augment the APT data in better characterizing the surface composition of NPG developed from electrolytic dealloying of Ag_95_Au_5_ alloys. Adsorption-desorption of HS^−^ in alkaline environments (0.2 M NaOH; pH 13) was found to be sufficiently selective towards Ag as opposed to Au, hence permitting quantification of the Ag surface coverage of the electrodes.

Cyclic voltammetry measurements on a pure polycrystalline Ag electrode in 0.2 M NaOH solutions containing 1 mM Na_2_S revealed a total of four peaks below the potentials required for bulk Ag_2_S formation. Measurements on a pure polycrystalline Au electrode showed a featureless capacitive current except for the region where HER is kinetically favorable.

In measurements on an as-annealed Ag_95_Au_5_ surface, four peaks appeared at similar potentials associated with HS^−^ electrosorption on Ag electrode, although each dampened in current density to a different extent. The faradaic charge densities associated with one peak at the half-wave potential of −0.37 V_Ag/Ag2S_ on the Ag_95_Au_5_ electrode was found to be very close to 95% Ag surface coverage. This peak, which was also not considerably affected by contributions from the HER on the electrodes used in this study, was then selected for calibration purposes.

The curves measured on NPG showed a featureless capacitive current except for the cathodic currents from the HER. Preliminary sensitivity analysis indicated that the peak used in calibration can be distinguished from the capacitive current densities, even when attenuated in intensity according to Ag surface coverages as low as 2%. Future work will focus on electrochemical characterization of NPG using other adsorbates and will further detail the sensitivity threshold by testing model alloys and known surface compositions.

Additionally, strong catalysis of water reduction was observed on the surface of pure Au and on NPG where sulfur was present in the solution.

## Data Availability

The data presented in this study are available upon reasonable request from the corresponding author. The data are not publicly available since they form parts of ongoing studies.
